# Association between relative free-living sit-to-stand transition thigh angular velocities and difficulties in mobility and daily functioning

**DOI:** 10.1007/s40520-026-03352-0

**Published:** 2026-02-26

**Authors:** Antti Löppönen, Kaisa Koivunen, Taina Rantanen, Laura Karavirta, Timo Rantalainen

**Affiliations:** Faculty of Sport and Health Sciences and Gerontology Research Center, University of Jyväskylä, PO Box 35, Jyväskylä, FI-40014 Finland

**Keywords:** Accelerometer, Relative intensity, Functional capacity

## Abstract

**Background:**

Sit-to-stand (STS) transitions are common daily activities, and we postulate that completing them with high effort relative to one’s capacity may indicate functional difficulties in older age.

**Aims:**

We examined the association between relative free-living STS thigh angular velocity and difficulties in activities of daily living (ADL) and instrumental activities of daily living (IADL), as well as walking and negotiating stairs.

**Methods:**

This cross-sectional study included 230 individuals (53% women) aged 79–89 years. STS capacity was assessed with an instrumented five-times STS test. Free-living STS transitions were monitored in 2-hour intervals over four days using an accelerometer. Associations were examined using Wilcoxon rank-sum test and logistic regression analyses.

**Results:**

In total, 47% had ADL/IADL difficulties, 26% reported walking difficulties, and 17% had difficulties negotiating stairs. Participants with any difficulties performed STS transitions at a higher proportion of their STS capacity compared to those without difficulties (*p* < 0.05). Higher relative free-living STS thigh angular velocity was associated with walking and stair negotiation difficulties in both crude (OR 1.23–1.53; *p* < 0.05) and adjusted models (OR 1.20–1.40; *p* < 0.05), while the association with ADL/IADL difficulties weakened after adjustment.

**Conclusions:**

Our findings suggest that performing STS transitions at high intensity may reflect emerging mobility difficulties.

**Supplementary Information:**

The online version contains supplementary material available at 10.1007/s40520-026-03352-0.

## Introduction

Certain daily activities, such as sit-to-stand (STS) transitions, are fundamental for maintaining independence, as daily tasks like using a chair or the toilet rely on the ability to perform successful STS transitions [1]. Notably, STS transitions are among the most common strength-demanding daily activities, and older adults may face challenges in executing them [2–4]. These difficulties are often related to an insufficient ability to produce enough force and movement to transition from a seated to a standing position efficiently [5], as well as balance impairments that can affect postural stability during the transition [6, 7].

STS transitions can be reliably detected and their velocity quantified in free-living environment using a single thigh-worn accelerometer (or inclinometer) [8–10]. Previous studies have assessed absolute STS velocity in a free-living environment based on thigh angular velocity [9, 10] and the duration of STS transitions [11, 12]. We have previously reported that absolute STS velocity may be a potential indicator of physical function decline in older adults [13]. However, examining free-living STS transitions velocity using absolute values may not fully capture how demanding they are for individuals with respect to their capacity. Moreover, every STS transition requires a minimum velocity to be successfully executed, which in turn depends on adequately coordinated momentum and torque generation [5]. Therefore, the thigh angular velocity of free-living STS transitions should be interpreted relative to an individual’s maximal capacity.

STS transition tests have been used to measure lower extremity strength and power output capacity in standardized environments [14, 15]. By instrumenting the test using wearable sensor technology, it is possible to isolate the STS phase, rather than relying solely on total execution time, which is a common outcome measure in tests such as the five-repetition STS test [16, 17]. This approach enables the measurement of thigh angular velocity during STS test (i.e., STS capacity), facilitating its direct comparison with free-living STS transitions. As a result, relative free-living STS thigh angular velocity can be calculated, accounting for an individual’s STS capacity and providing a more personalized assessment.

We hypothesise that having to perform daily activities at high relative intensity may pose a challenge to one’s ability to maintain independence [18], i.e., sufficient capacity reserve is necessary in order to cope with the requirements of independent living. We address the hypothesis by examining how free-living STS thigh angular velocity expressed as a percentage of individual STS capacity is associated with activities essential for independent living. Unlike previous studies based on short laboratory protocols [19], our study captures STS transitions continuously in a free-living environment over 24 h for five consecutive days, providing a more ecologically valid perspective than a single structured assessment. Because device-based methods enable the assessment of diurnal trends [20, 21], we examined this association using 2-hour intervals to determine whether daily variation in relative free-living STS velocity exists and to understand its potential implications for functional capacity throughout the day. The purpose of the present study was to investigate how the velocity of STS transitions in a free-living environment across the day, relative to individual capacity, is associated with difficulties in basic and instrumental daily activities (ADL/IADL), walking difficulties, and difficulties negotiating stairs in community-dwelling older adults. If confirmed, these findings would have clinical implications by helping to identify individuals at risk of functional decline and guiding interventions to maintain independence.

## Methods

### Participants and design

The data for this cross-sectional study were obtained from the AGNES study follow-up (Active Aging―Resilience and External Support as Modifiers of the Disablement Outcome; baseline *n* = 1,021), conducted at the Gerontology Research Center, University of Jyväskylä, Finland. At baseline, 479 participants comprised the accelerometer subsample with valid data for at least three full days [22]. The data for this study were obtained from the second-wave measurements of the AGNES study. The sample included 679 participants from three age cohorts (79, 84, and 89 years) who were independently living in the city of Jyväskylä, located in Central Finland [23]. The measurements were carried out in 2021–2022. In this follow-up, participants who had been part of the baseline accelerometer subsample were invited again to wear an accelerometer. Of these, 230 participants (53% women) with at least three days of free-living accelerometer data and an instrumented 5 × STS test were included in the present analysis. The Ethical Committee of the Central Finland Health Care District provided an ethical statement on the research plan and protocol of the AGNES measurements (September 8, 2021). The study was executed in accordance with the principles of the Declaration of Helsinki. All participants provided written informed consent.

### Relative free-living STS assessment

Free-living STS transitions were monitored using a thigh-worn accelerometer (UKK RM42 tri-axial accelerometer, 100 Hz, 13-bit analog-to-digital conversion, acceleration range ± 16 g, UKK Health Services Ltd, Tampere, Finland) continuously (24 h) for 4 consecutive days from midnight to midnight. Free-living STS transitions were evaluated from the accelerometer data using our open-access algorithm (https://github.com/tjrantal/SitToStandSupplement) that detects STS transitions and quantifies the thigh angular velocity of the movement. The full structure of the algorithm, together with all thresholds, implementation details and operational properties, is presented elsewhere [8, 9]. The quantification of thigh angular velocity during free-living STS transitions by the algorithm aligns with the results of 2-D motion analysis and achieved a detection accuracy of over 93% for identifying STS transitions in a similar population of older adults [9].

The STS capacity was assessed at participants’ homes using the five-time STS test (5×STS), which is a part of the Short Physical Performance Battery (SPPB), along with walking speed and standing balance tests. Each test is scored from 0 to 4, with a total SPPB score ranging from 0 to 12, where higher scores indicate better physical functioning [15]. The STS capacity variable, representing thigh angular velocity during the 5×STS test, was derived from the sensor recordings and calculated as the median value across the five repetitions to account for potential repetition-level outliers [16].The test was performed on an armless chair with participants’ hands crossed over the chest, and hand use was not permitted. The data that included the STS test occurred before the first midnight of the recording and was disregarded in the free-living STS analyses. Free-living STS transitions were grouped into two-hour intervals based on their real-time timestamps (e.g., 08:00–10:00, 10:00–12:00), and the median number of transitions within each interval was calculated. A daily median across all monitoring days was then computed to obtain the final summary measure. The relative STS velocity was determined by dividing the velocity of each free-living STS transition by each participant’s individual STS capacity. As the number of STS transitions was low during nighttime, this study focused on daytime STS behaviour between 08:00 and 22:00 in two-hour time intervals. The 24-hour trends are presented in Supplementary File 1.

### Activities of daily living, walking difficulties, and difficulties in negotiating stairs

Activities of daily living was evaluated using a twelve-item questionnaire [24]. Basic ADLs (ADL) encompass tasks such as feeding, getting up from or lying down on a bed, dressing, bathing, and toileting. Instrumental ADLs (IADL) comprise activities like meal preparation, shopping, light household chores (e.g., washing dishes), heavier household chores (e.g., sweeping floors), medication management, handling finances, and utilizing public transportation. Participants rate their ability to perform each task on a scale from one (able without difficulty) to five (unable even with assistance from another person) [24]. Due to the limited number of ADL difficulties observed, ADL and IADL issues were combined into a single “*ADL/IADL difficulties*” to enhance the robustness of analyses when individual ADL data is sparse [25, 26]. The ADL/IADL difficulties variable was defined as dichotomous: “No difficulties,” indicating no difficulties in any ADL/IADL function, and “Yes difficulties,” indicating difficulties at any level or in any ADL/IADL activity.

Self-reported walking difficulties were assessed for covering a 2-km distance, using standardized questions commonly applied in mobility research, which reflects the relatively high functional level of this study sample [27, 28]. Participants were asked about their perceived difficulty in walking 2 km with the question: ‘Do you have difficulty walking 2 km?’ The response options included: “able to manage without difficulty”, “able to manage with some difficulty”, “able to manage with a great deal of difficulty”, “able to manage only with help of another person” and “unable to manage even with help”. For this study, responses were categorized as “No difficulties” or “Yes difficulties,” with all options other than “able to manage without difficulty” merged into the latter.

Difficulties in negotiating stairs were assessed with the question: “Have you noticed any changes in your ability to ascend a flight of stairs?” The response options included: “No difficulties,” “I can ascend a flight of stairs, but I have some difficulties,” “I can ascend a flight of stairs, but I have a lot of difficulties,” “I cannot ascend a flight of stairs without the help of another person,” and “I cannot ascend a flight of stairs even with help.” [23]. For this study, responses were categorized as “No difficulties” or “Yes difficulties,” with all options other than “No difficulties” merged into the latter. Notably, no participants reported “I cannot ascend a flight of stairs even with help.”

### Descriptive characteristics and other measurements

Age and sex were extracted from the Digital and Population Data Services Agency in Finland. Socioeconomical status was measured with self-reported years of education [23]. Cognitive function was assessed with the Mini-Mental State Examination (MMSE) which contains 19 items and the score ranges from 0 to 30, with higher values indicating better cognitive functioning [29], and depressive symptoms were assessed using the Center for Epidemiologic Studies Depression Scale (CES-D), a 20-item self-report questionnaire with a total score ranging from 0 to 60, where higher scores indicate more severe depressive symptoms [30]. Handgrip strength was measured with a hand-held adjustable dynamometer (Jamar Plus digital hand dynamometer, Patterson Medical, Cedarburg, WI, USA), and expressed in kg [31].

### Statistical analyses

Descriptive data are presented as means and standard deviations (SD). The Shapiro-Wilk test and visual inspection indicated that the data were not normally distributed; therefore, non-parametric methods were used for the group comparisons. Independent-Samples Mann-Whitney U (Wilcoxon rank-sum) test for group comparisons and the chi-square test was applied for analysing categorical variables. The Mann-Kendall trend test was applied separately to each group to assess monotonic trends in sit-to-stand transition speeds, with tau and p-values used to determine the direction and significance of the trend over time. The statistical difference in absolute and relative STS thigh angular velocity between groups with ADL/IADL difficulties, walking difficulties, and difficulties negotiating stairs was assessed using the Independent-Samples Mann-Whitney U (Wilcoxon rank-sum) test.

Logistic regression was used to assess the effect size (Odds Ratio = OR and 95% confidence interval) of relative STS thigh angular velocity between groups with ADL/IADL difficulties, walking difficulties, and difficulties negotiating stairs [32]. We conducted two models: a crude model and an adjusted model. The adjusted model controlled for age, sex, cognitive function (MMSE), depressive symptoms (CES-D), and hand grip strength. Depressive symptoms are associated with ADL limitations and gait performance and may influence both self-reported difficulties and physical performance [33]. Cognitive function is a known confounder for functional outcomes and mobility [34, 35]. Hand grip strength was included to account for overall muscle strength, an important determinant of physical function and mobility. Statistical significance was set at *P* < 0.05. Effect sizes were interpreted using odds ratios (OR) from logistic regression, with thresholds defined as 1.5 for small, 2.5 for medium, and 4.0 for large effects [32]. Odds ratios are reported per 10% increase in relative STS velocity. The analyses presented in the figures were conducted using the R statistical environment (version 4.2.1) [36], while group comparisons in descriptive statistics and logistic regression analyses were performed using the SPSS statistical software package (IBM SPSS Statistics Version 28.0.1.1; SPSS Inc., Chicago, IL) [37].

## Results

Descriptive characteristics are presented in Table 1. Among participants, 47% had ADL/IADL difficulties, 26% reported walking difficulties, and 17% had negotiating stairs difficulties. Participants reporting mobility-related difficulties (ADL/IADL, walking, or negotiating stairs) were older and had lower SPPB scores, grip strength, and STS capacity, and those with ADL/IADL and 2-km walking difficulties had higher depressive scores (*p* < 0.05) compared to those with no reported difficulties.

**Table 1:** Descriptive characteristics of the participants (mean ± standard deviation (SD))

	ADL/IADL difficulties			2km walking difficulties				Difficulties negotiating stairs		
	No	Yes		No	Yes			No	Yes	
	n = 122	n = 108		n = 171	n = 59			n = 191	n = 39	
Sex [Women %]	50 %	57 %	0.326	52 %	56 %	0.606		50 %	67 %	0.061
Age [years]	81.2 ±2.8	82.3 ±3.2	0.004	81.4 ±3.0	82.6 ±3.0	0.009		81.4 ±2.9	83.1 ±3.4	0.005
CES-D [points]	8.0 ±5.7	10.2 ±6.4	0.010	8.1 ±5.5	11.9 ±7.0	<0.001		8.7 ±5.8	10.9 ±7.3	0.089
MMSE [points]	27.8 ±2.1	27.1 ±2.7	0.108	27.6 ±2.3	27.2 ±2.9	0.725		27.6 ±2.2	26.7 ±3.4	0.415
SPPB total score [points]	10.8 ±1.3	9.8 ±1.9	<0.001	10.7 ±1.4	9.4 ±2.0	<0.001		10.6 ±1.5	9.0 ±1.9	<0.001
Handgrip force [kg]	33.1 ±9.5	28.6 ±9.1	<0.001	32.0 ±9.7	27.9 ±8.5	0.007		32.0 ±9.6	26.0 ±7.9	<0.001
STS capacity [deg/s]	122.4 ±20.2	107.7 ±22.7	<0.001	119.7 ±21.4	103.2 ±21.7	<0.001		119.1 ±21.6	97.9 ±19.1	<0.001

CES-D = Center for Epidemiologic Studies Depression Scale, MMSE = Mini-Mental State Examination, SPPB = Short Physical Performance Battery, STS = sit-to-stand .P-values: Groups differences were combined and analyzed using the Independent-Samples Mann-Whitney U (Wilcoxon rank-sum) test and dichotomous sex differences were analyzed using the Chi-Square test.

**Fig. 1:**
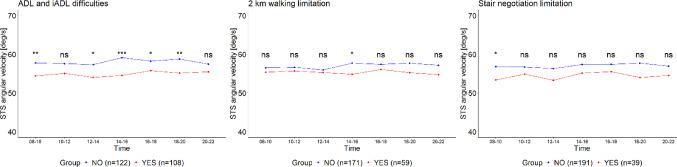
Absolute mean angular velocities of STS transitions among older adults with and without ADL/IADL, walking, and stair-climbing difficulties, analyzed across 2-hour time intervals during daytime (08-22). Note: Group differences at each time point were analyzed using the Mann-Whitney U (Wilcoxon rank-sum) test. Statistical significance is indicated by asterisks: ***p < 0.001, **p < 0.01, *p < 0.05, and 'ns' denotes non-significant results

**Fig. 2:**
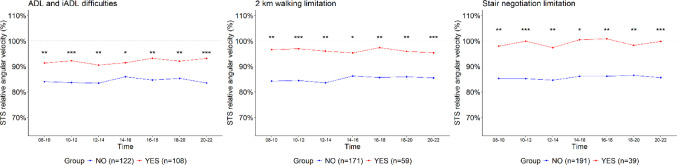
Relative thigh angular velocities of STS transitions with and without ADL/IADL, walking, and stair-climbing difficulties, analyzed across 2-hour time intervals during daytime (08-22). Note: Group differences at each time point were analyzed using the Mann-Whitney U (Wilcoxon rank-sum) test. Statistical significance is indicated by asterisks: ***p < 0.001, **p < 0.01, *p < 0.05, and 'ns' denotes non-significant results

Participants reporting ADL/IADL difficulties had lower absolute free-living STS thigh angular velocity compared to those who did not report such difficulties, except in two time intervals (10:00–12:00 and 20:00–22:00) (Fig. 1). A similar difference was not observed between those reporting walking or difficulties negotiating stairs (*p* < 0.05), except in one time interval (14:00–16:00 and 08:00–10:00, respectively) (Fig. 1). However, relative free-living STS thigh angular velocity differed between participants reporting difficulties in ADL/IADL functions, walking, or negotiating stairs, such that those experiencing difficulties used a larger proportion of their capacity during free-living STS transitions compared to participants without such difficulties (all *p* < 0.05) (Fig. 2).

Higher relative free-living STS thigh angular velocity was associated with having walking difficulties and difficulties negotiating stairs in the crude model (Odds Ratio (OR) 1.23–1.53, *p* < 0.05) and in models adjusted for age, sex, cognitive performance, depression scores and handgrip strength (OR 1.20–1.40, *p* < 0.05). In the crude model, higher relative free-living STS thigh angular velocity was also associated with ADL/IADL function difficulties (OR 1.16–1.29, *p* < 0.05). In the adjusted model, this association was attenuated at certain time periods (08:00–10:00, 14:00–16:00, and 18:00–20:00) but remained at the other assessed periods (Table 2).

**Table 2:** Relative STS velocity and its association with ADL/IADL difficulties, walking difficulties, and negotiating stairs difficulties, presented as odds ratios (OR) with 95% confidence intervals

Time interval	08–10	10–12	12–14	14–16	16–18	18–20	20–22
ADL/IADL difficulties							
Relative STS velocity [decrease 10%] crude	**1.23** **(1.07–1.43)**	**1.29** **(1.12–1.50)**	**1.19** **(1.05–1.37)**	**1.16** **(1.01–1.32)**	**1.27** **(1.10–1.46)**	**1.22** **(1.06–1.41)**	**1.26** **(1.10–1.44)**
Relative STS velocity [decrease 10%] adjusted	1.15 (0.97–1.33)	**1.20** **(1.03–1.41)**	**1.16** **(1.01–1.33)**	1.09 (0.94–1.26)	**1.19** **(1.02–1.38)**	1.13 (0.97–1.32)	**1.18** **(1.02–1.36)**

Walking difficulties							
Relative STS velocity [decrease 10%] crude	**1.43** **(1.21–1.68)**	**1.44** **(1.21–1.71)**	**1.38** **(1.17–1.61)**	**1.26** **(1.09–1.47)**	**1.36** **(1.16–1.59)**	**1.34** **(1.13–1.57)**	**1.23** **(1.07–1.41)**
Relative STS velocity [decrease 10%] adjusted	**1.32** **(1.11–1.58)**	**1.35** **(1.13–1.62)**	**1.33** **(1.12–1.57)**	**1.20** **(1.02–1.41)**	**1.29** **(1.09–1.53)**	**1.24** **(1.04–1.48)**	1.16 (0.99–1.34)

Difficulties negotiating stairs							
Relative STS velocity [decrease 10%] crude	**1.41** **(1.18–1.71)**	**1.53** **(1.26–1.85)**	**1.38** **(1.15–1.65)**	**1.44** **(1.20–1.73)**	**1.45** **(1.21–1.73)**	**1.39** **(1.16–1.68)**	**1.32** **(1.13–1.54)**
Relative STS velocity [decrease 10%] adjusted	**1.27** **(1.03–1.55)**	**1.40** **(1.14–1.72)**	**1.30** **(1.08–1.57)**	**1.35** **(1.11–1.64)**	**1.32** **(1.09–1.60)**	**1.32** **(1.07–1.62)**	**1.26** **(1.06–1.49)**

Crude model: No adjustments. Adjusted model: Adjusted for age, sex, cognitive functioning (MMSE), depression scores and hand grip strength. Bolded OR values indicate statistical significance (*p* < 0.05).

The absolute STS velocity trends throughout the day did not differ between individuals without ADL/IADL function difficulties, walking or negotiating stairs (*p* = 0.76, *p* = 0.37, and *p* = 0.23, respectively), nor among those experiencing these difficulties (*p* = 0.13, *p* = 0.76, and *p* = 0.55, respectively) (Fig. 1). Similarly, the relative STS velocity trends throughout the day showed no differences between individuals without (*p* = 0.23, *p* = 0.37, and *p* = 0.76) and with difficulties (*p* = 0.76, *p* = 0.37, and *p* = 0.23) (Fig. 2).

## Discussion

This study examined if free-living STS thigh angular velocity expressed as a percentage of individual STS capacity were associated with difficulties in activities of daily living (ADLs/IADLs), 2 km walking, and negotiating stairs among community-dwelling older adults. The results indicate that the relative free-living STS velocity was higher in individuals with self-reported functional difficulties compared to those without. Specifically, individuals with difficulties perform free-living STS transitions in a free-living environment closer to their STS capacity, while those without difficulties exhibit a greater difference between their free-living STS velocity and their STS capacity. The analysis of diurnal trends revealed that the association remained consistent throughout the day, indicating that differences in relative free-living STS velocity between individuals with and without functional limitations reflect a stable feature of daily functional capacity rather than time-specific effects. These findings highlight the importance of maintaining sufficient physical capacity. Although individuals may still be able to perform daily activities, doing so at high relative intensity suggests a diminishing physical reserve and reflects emerging difficulties with mobility.

We have previously observed that older age groups and individuals with lower SPPB scores perform free-living STS transitions at a higher proportion of their STS capacity compared to younger and those with higher SPPB scores among middle-aged and older adults [16]. The findings of this study are aligned with our previous observations while extending them by demonstrating significant difficulties in self-reported difficulties in ADL/IADLs, as well as walking and negotiating stairs. Notably, relative free-living STS thigh angular velocity showed greater differences between individuals with and without stair negotiation difficulties than between those with and without ADL/IADL difficulties. This may be due to the fact that older adults who report difficulties in negotiating stairs often represent a subgroup with more pronounced functional difficulties and overall lower physical capacity compared to those with only ADL/IADL difficulties [38].

We scaled the thigh angular velocity of free-living STS transitions to individual STS capacity to derive relative STS thigh angular velocity. The findings showed that absolute free-living STS thigh angular velocity differences between participants with and without difficulties in activities of daily living (ADLs/IADLs), walking, or negotiating stairs were small and not statistically significant at any time of day. This suggests that STS capacity is a key determinant of how closely older adults perform free-living STS transitions to their maximum ability. Therefore, maintaining capacity is crucial, as it defines the upper limit for functional ability and plays a central role in enabling daily activities without difficulty [39] However, the optimal free-living STS transition velocity remains unknown, particularly given variations in living environments. For example, assisted living facilities often provide optimized chair heights, whereas typical homes may lack an ideal setup for individuals with functional difficulties [40] Future research should investigate whether a theoretical optimal velocity exists for free-living STS transitions that supports efficient and safe STS transitions. In addition, the 5×STS reflects maximal STS capacity under safe and structured conditions, whereas free-living performance represent related but distinct constructs [41, 42], and includes uncontrollable elements related to environment and behaviour. Laboratory-based capacity may set upper limits for, but does not fully determine, STS performance in free-living environments.

The gap between absolute free-living STS thigh angular velocity and STS capacity, that is, relative STS thigh angular velocity, may reflect an individual’s physical reserve. When reserve is low, free-living STS transitions are performed closer to one’s STS capacity, increasing the likelihood of self-reported difficulties. Physical reserve has been suggested to act as a buffer against negative outcomes following accidents [43, 44] and is therefore a crucial factor in supporting the independence of older adults living in the community. Importantly, this reserve must also be sufficient across different times of the day, as fatigue-related reductions in functional capacity toward the evening may compromise task performance and increase vulnerability to adverse events, such as falls. However, as mentioned above, defining physical reserve through STS transitions would require a more structured understanding of the minimum requirements for STS transitions across individuals. Although free-living STS performance was associated with ADL/IADL function and perceived difficulty in mobility tasks, the cross-sectional design prevents determining the direction of these associations. This issue should be examined in future longitudinal studies, ideally using cross-lagged models to clarify temporal ordering and potential causal pathways.

We present an approach for measuring 5×STS capacity that builds on earlier instrumented methods [17], focusing on the velocity of the sit-to-stand phase rather than total test time. We extend this approach by applying the same metric to free-living STS transitions and by deriving a relative angular-velocity measure that links laboratory-based capacity with free-living performance. This integration enables tracking changes in both controlled and free-living environments and supports continuous monitoring of functional progress, for example during rehabilitation. One potential clinical application could be following recovery after events such as hip fracture surgery by tracking everyday STS performance relative to an individual’s maximum capacity (pre-operation). Furthermore, studies should compare the predictive validity of this metric with established tests such as the 5xSTS and the 10-meter walk test to determine its added clinical value and whether continuous free-living monitoring provides additional benefits beyond traditional assessments.

Several limitations should be considered when interpreting the findings of this study. First, while the sample size is relatively large, participation in device-based measurements tends to attract a selective group of individuals, which may restrict the generalizability of the findings. There is evidence that participants in device-based studies were more active, as the proportion classified in the most active category increased notably during the monitoring week [45]. Second, although determining strength-demanding activities through STS transitions provides valuable insights, it does not encompass other strength-demanding activities such as negotiating stairs or squatting down and rising back up. Additionally, STS transitions, which can be identified with over 93% accuracy [8], are well-suited for the purpose of this study, but we were unable to determine whether participants used their hands during transitions. Although prior biomechanical evidence suggests that hand support does not necessarily increase transition speed [46, 47], factors such as fear of falling or perceived instability may still influence movement velocity in free-living conditions. Furthermore, collapsing the difficulty scales into two categories, although necessary to retain adequate group sizes in this high-functioning sample, reduced the granularity of the outcome measures. Finally, other factors such as pain could not be taken into account. Despite these limitations, the study has notable strengths. The multi-day continuous 24-hour monitoring period enables a robust evaluation of physical activity behaviours [48]. Free-living STS transitions are particularly relevant activities as they are essential for independent living, and some of them such as standing up to use the toilet are driven by necessity rather than personal motivation or leisure preferences, unlike activities such as moderate-to-vigorous physical activity. Furthermore, the study recruited a relatively large sample of community-dwelling older adults, allowing for a detailed examination of this population group.

## Conclusions

These findings suggest that higher proportion of free-living STS angular velocity relative to individual STS capacity, is associated with difficulties in activities essential to independent living. Continuous monitoring of STS performance in free-living environments may provide additional insights beyond traditional assessments and could support future applications in rehabilitation and early detection of functional decline. However, longitudinal studies are required to determine whether this metric predicts future functional decline, adds predictive value beyond established tests, and offers meaningful clinical benefits over time.

## Supplementary Information

Below is the link to the electronic supplementary material.

Supplementary Material 1

## Data Availability

After completion of the study, data will be stored at the Finnish Social Science Data Archive without potential identifiers (open access). Until then, pseudonymized datasets are available to external collaborators subject to agreement on the terms of data use and publication of results. To request the data, please contact Professor Taina Rantanen (taina.rantanen@jyu.fi).
