# Supplementary figures and images for: Association between relative free-living sit-to-stand transition thigh angular velocities and difficulties in mobility and daily functioning
Source: Aging Clin Exp Res. 2026 Feb 26;38(1):101. doi: 10.1007/s40520-026-03352-0 (PMC13005777; doi:10.1007/s40520-026-03352-0)

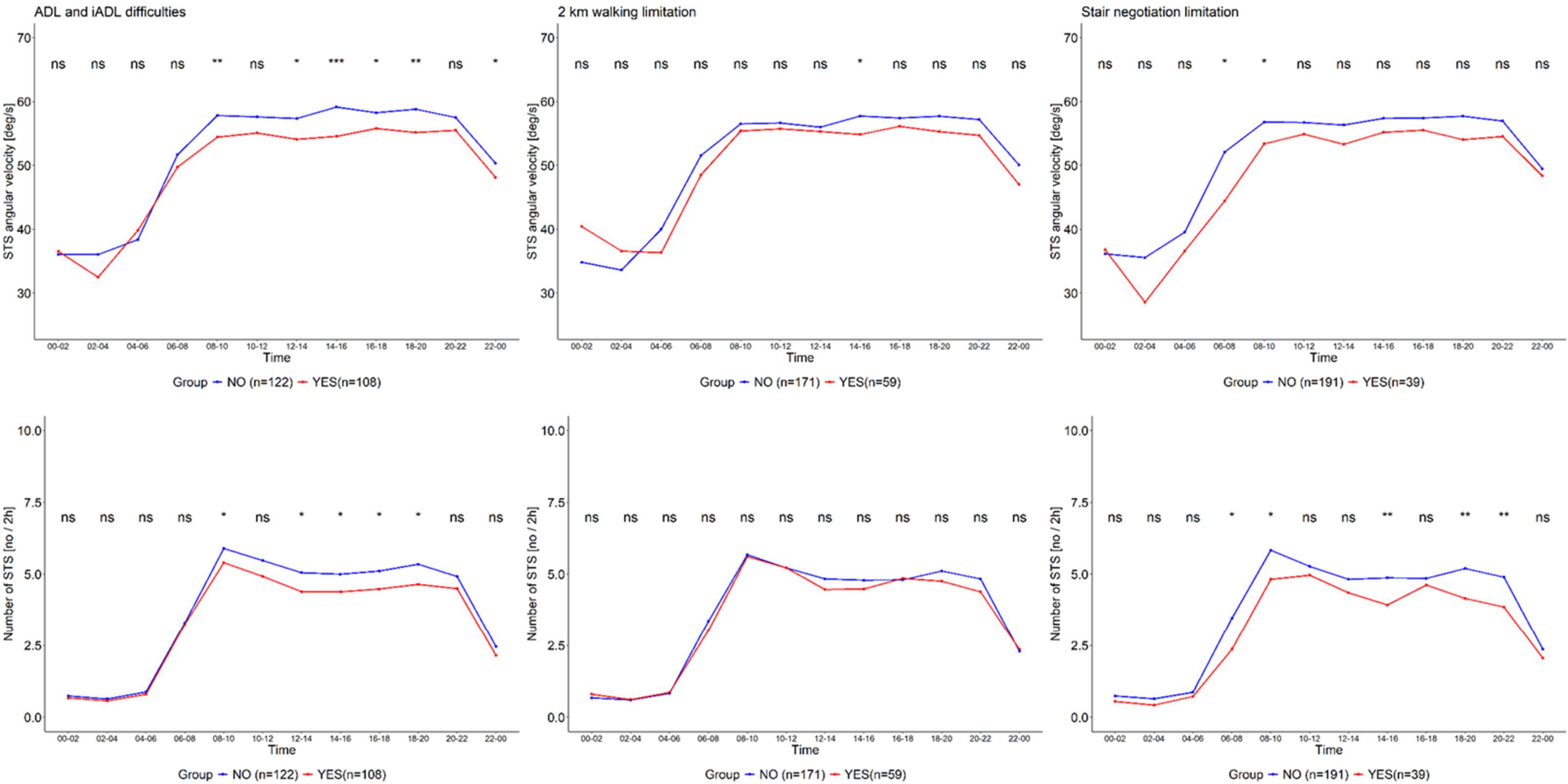

Supplement: Supplementary file 1 — Supplementary Material 1 [file 40520_2026_3352_MOESM1_ESM.pdf]
